# Cardinal Points in Bacteriology

**Published:** 1891-08

**Authors:** 


### Cardinal Points in Bacteriology.

The *Bacteriological World* says: The words germ, bacteria, microbe schizomycetes are used in our present literature almost as synonymous terms, but microbe seems preferable to germ or bacteria, and schizomycetes is a better scientific term than either.

That these are unicellular and assimilate nourishment seemingly by absorption in the media in which they live, but they must transform (alter) the foods found proper, and yet unfit in nature, for their use and appropriation.

Bacteria living on dead matter encounter no living resistance, whilst those feeding on living tissues, or fluids in living tissues, meet the living cells of the body and have to combat them.

The diastases secreted by the various beings, whether highly organized, or unicellular and microscopic have something in common as to their respective objects and their properties of transforming matter.

The role of microbes in the world is complex and necessary, though some are injurious. They act as scavengers, return to the air and water the organizable elements abstracted daily by the vegetables of the globe, and indirectly by animals, and indispensable to life.

The bacteria that invade living organisms which happen to be fit for their nourishment and growth are, in a sense, parasites just as much as the tapeworm is.

Spontaneous generation of living organisms, no matter how little, is a fallacy.

Increase in the Use of Alcohol in France.—From late returns it is found that the consumption of alcohol in France is largely increasing, and this despite the fact of the decrease in population. Can it be shown that there is a relation between these two processes?—*The Journal.*

